# Supplementary material for: A beneficial endornavirus enhances the fitness of the phytopathogenic fungus Rhizotonia solani
Source: mBio. 2026 Apr 20;17(5):e00166-26. doi: 10.1128/mbio.00166-26 (PMC13170352; doi:10.1128/mbio.00166-26)
Supplement: Supplemental Material — Fig. S1-S16; Tables S1 and S2. [file mbio.00166-26-s0003.docx]

Supplementary Materials for

**A Beneficial Endornavirus Enhances the Fitness of the Phytopathogenic Fungus *Rhizotonia solani***

Tianxing Pang^1^, Bokang Li^1^, Qianqian Sun^1^, Zhiping Deng^2^, Chunmei Cao^3^, Zhensheng Kang ^a^, Ida Bagus Andika^1^*, Liying Sun^1, 4^

1. State Key Laboratory of Crop Stress Biology for Arid Areas and College of Plant Protection, Northwest A&F University, Yangling 712100, Shaanxi, China
2. Institute of Virology and Biotechnology, Zhejiang Academy of Agricultural Sciences, Hangzhou 310021, Zhejiang, China
3. Potato Research Center, Inner Mongolia Academy of Agricultural &Animal Husbandry Sciences, 010031 Hohhot, China
4. Institute of Future Agriculture, Northwest A&F University, Yangling 712100, Shaanxi, China

***Corresponding author**

Ida Bagus Andika

E-mail: ibandika@nwafu.edu.cn

Liying Sun

E-mail: sunliying@nwafu.edu.cn

Phone no.: 86-29-87082477

Fax no.: 86-29-87082477

**This PDF file includes:**

Supplementary Text

Figs. S1 to S16

Tables S1 to S2

**Other Supplementary Materials for this manuscript include the following:**

Data files S1 to S2

**
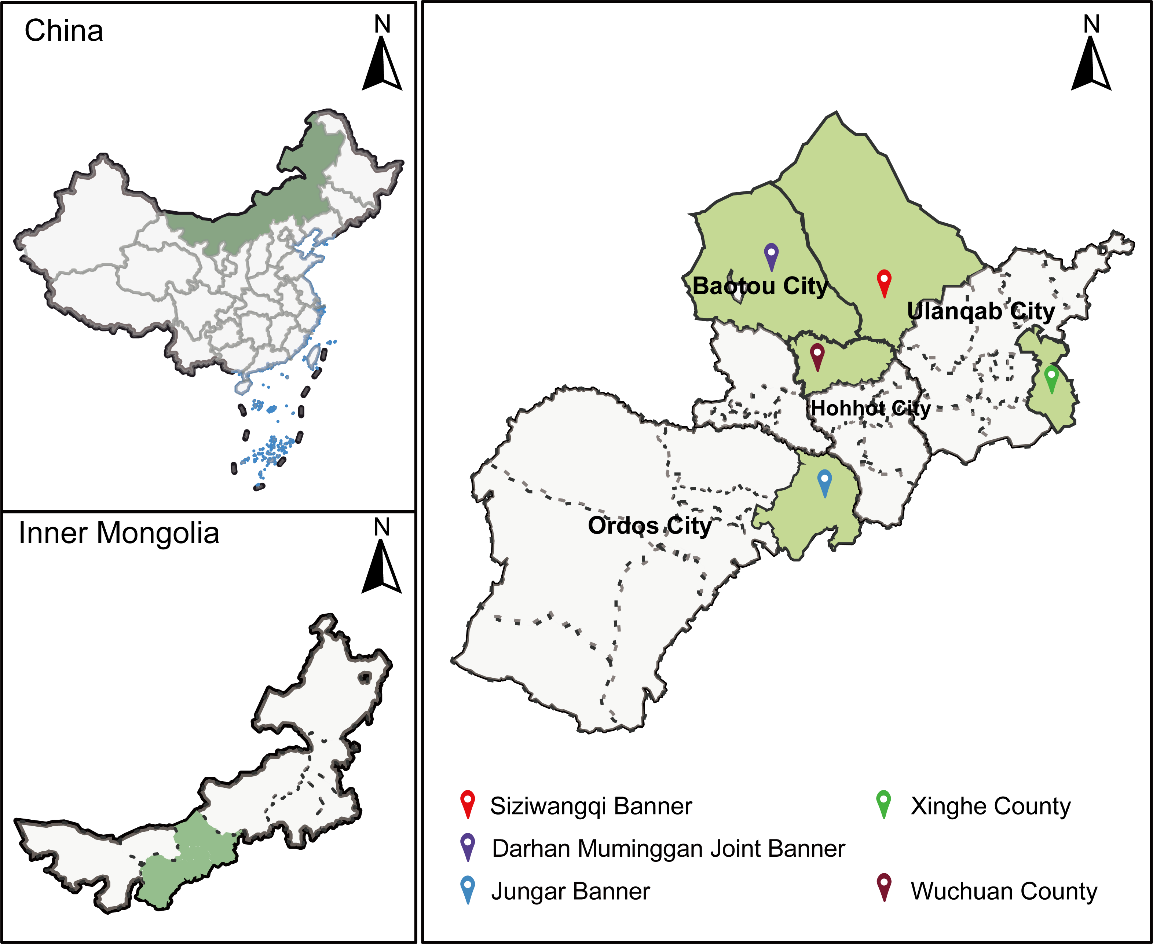
**

**Figure S1.**Maps showing the locations where potato samples were collected for the isolation of *R. solani* strains.


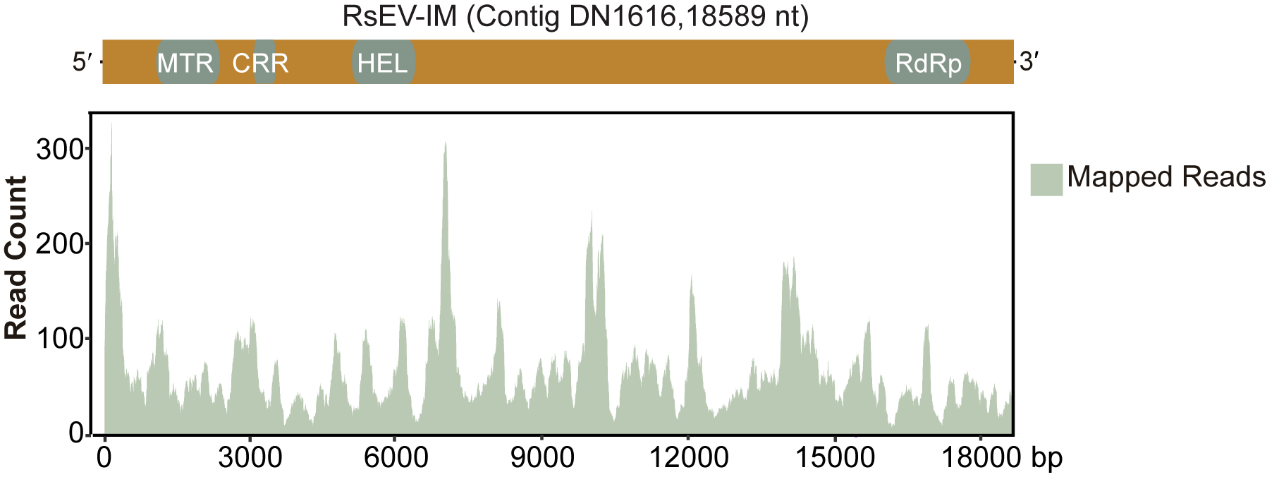


**Figure S2**. Reads mapped to an endornavirus-related contig.


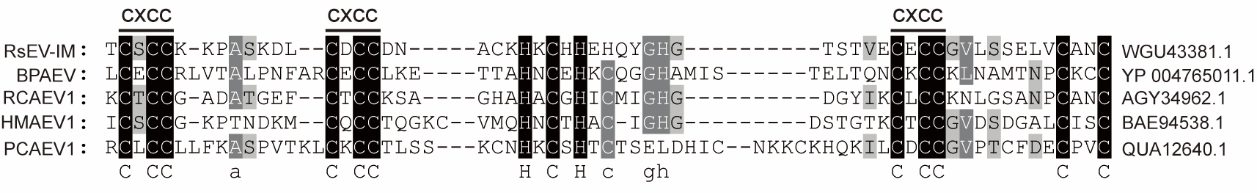


**Figure S3**. Putative cysteine-rich region (CRR) with conserved "CXCC" signature sequences found in RsEV-IM and other endornaviruses.


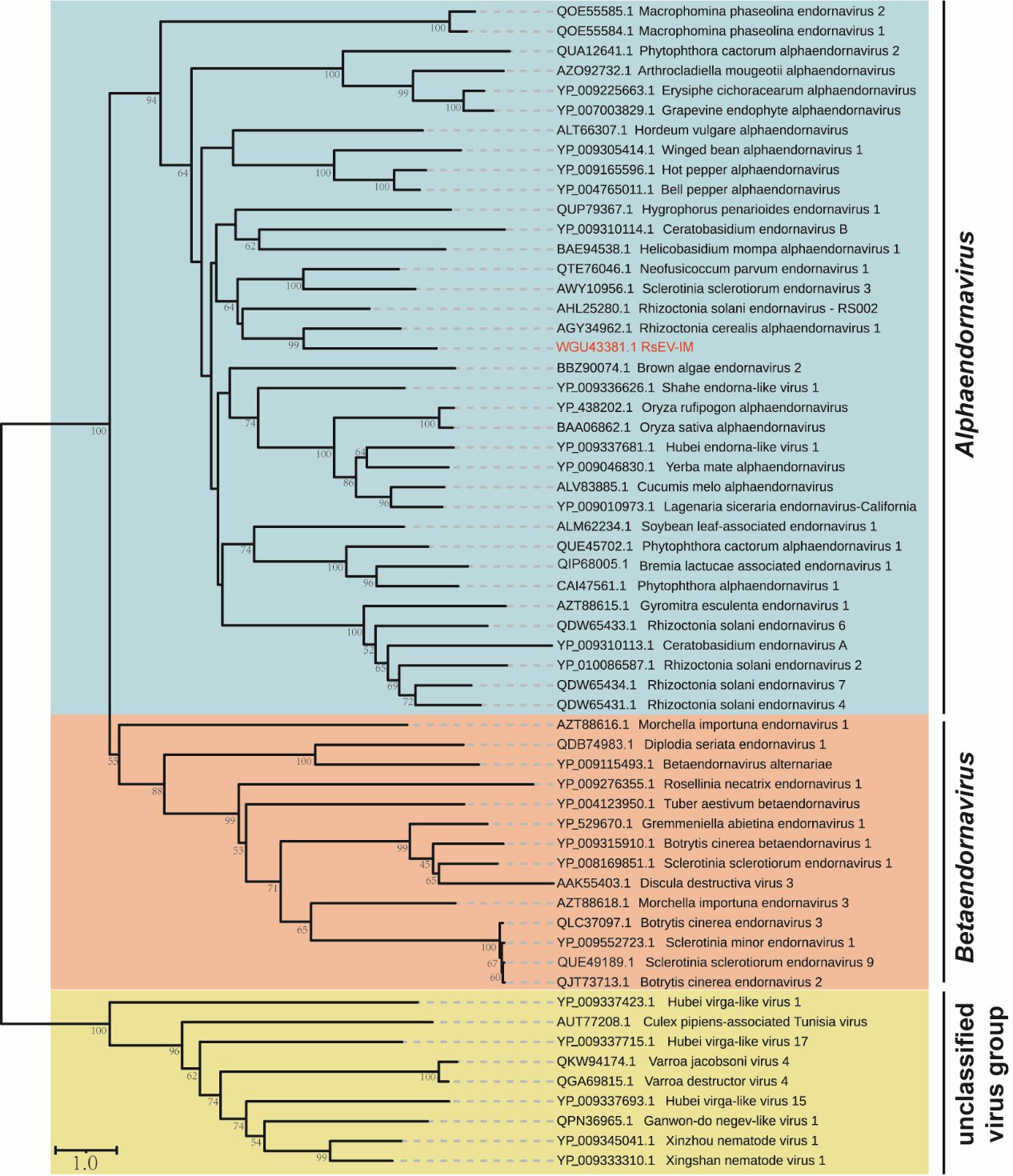


**Figure S4**. Phylogenetic relationships of RsEV-IM to endornaviruses and selected endorna-like viruses. The tree was constructed using a maximum-likelihood method (JTT matrix-based model) based on a multiple sequence alignment of the RdRp domain. Branch numbers indicate the percentage of replicate trees in which the associated taxa clustered together. The tree is drawn to scale, with branch lengths representing the number of substitutions per site. Virus names are preceded by their accession numbers.


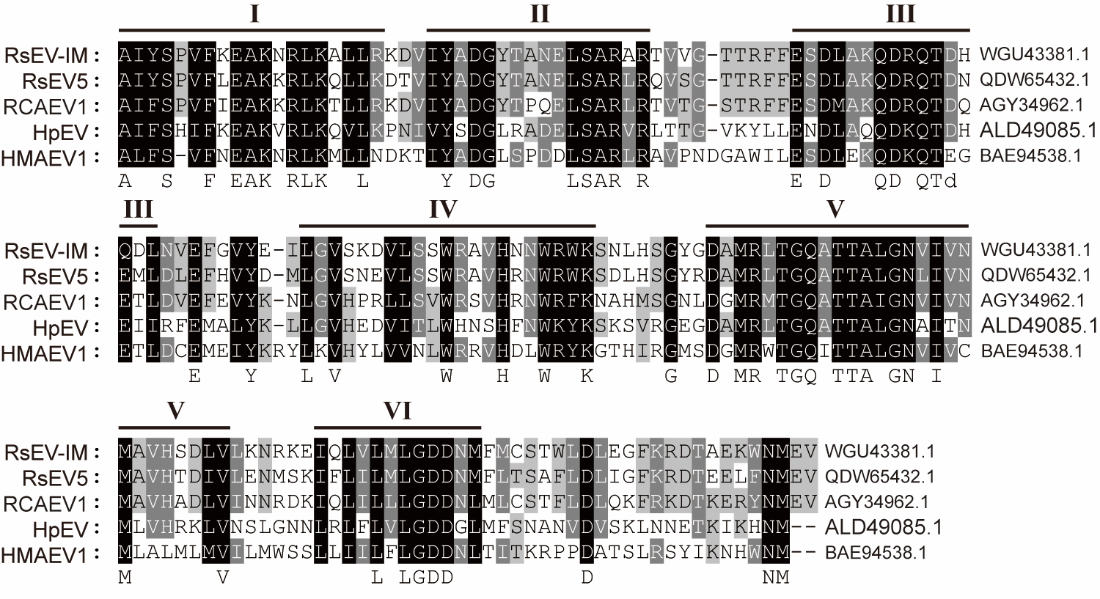


**Figure S5**. Multiple sequence alignment of the RdRp domain of RsEV-IM with those of alphaendornaviruses.


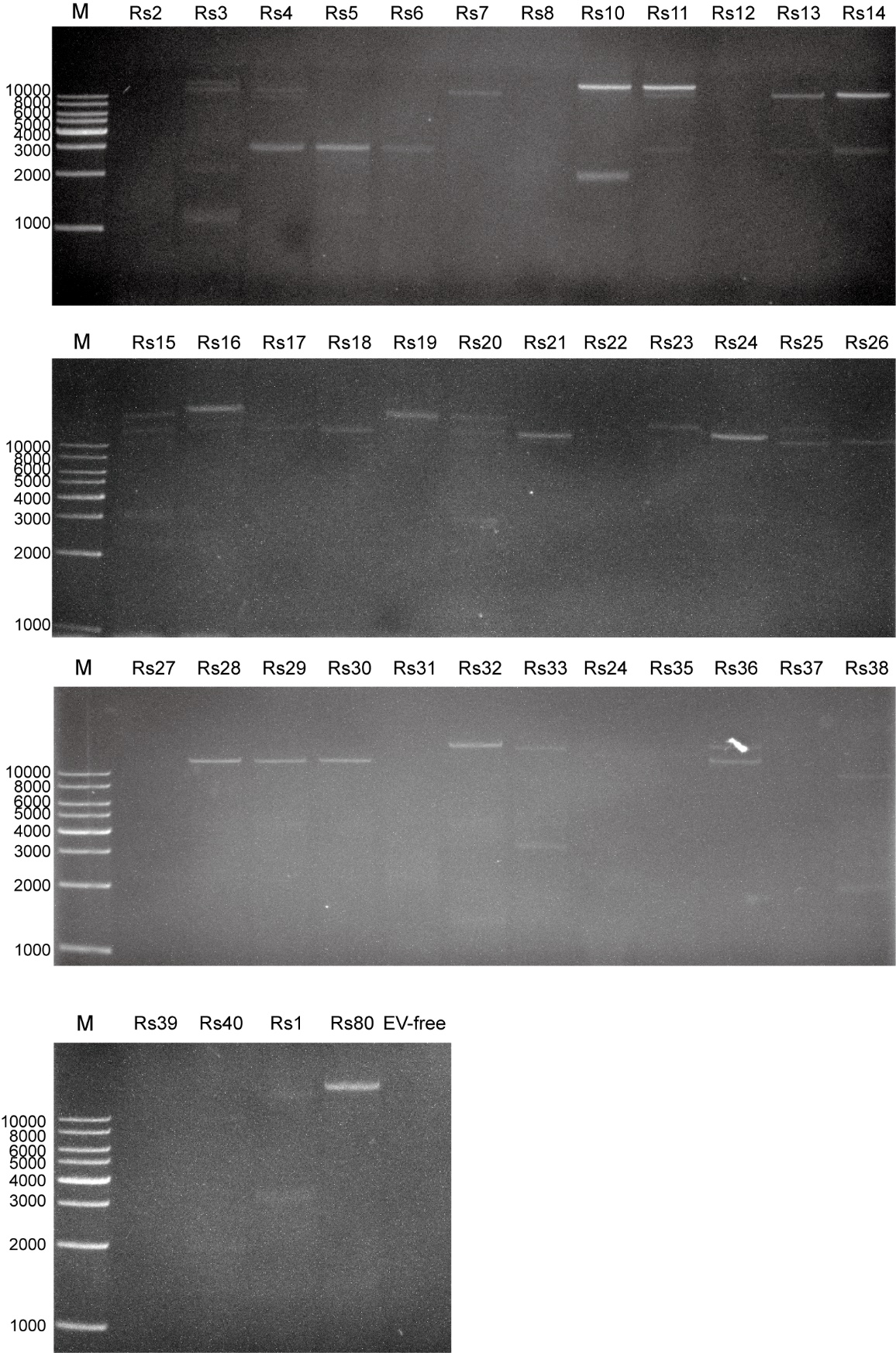


**Figure S6**. Profiles of dsRNA extracted from *R. solani* AG-3 strains.


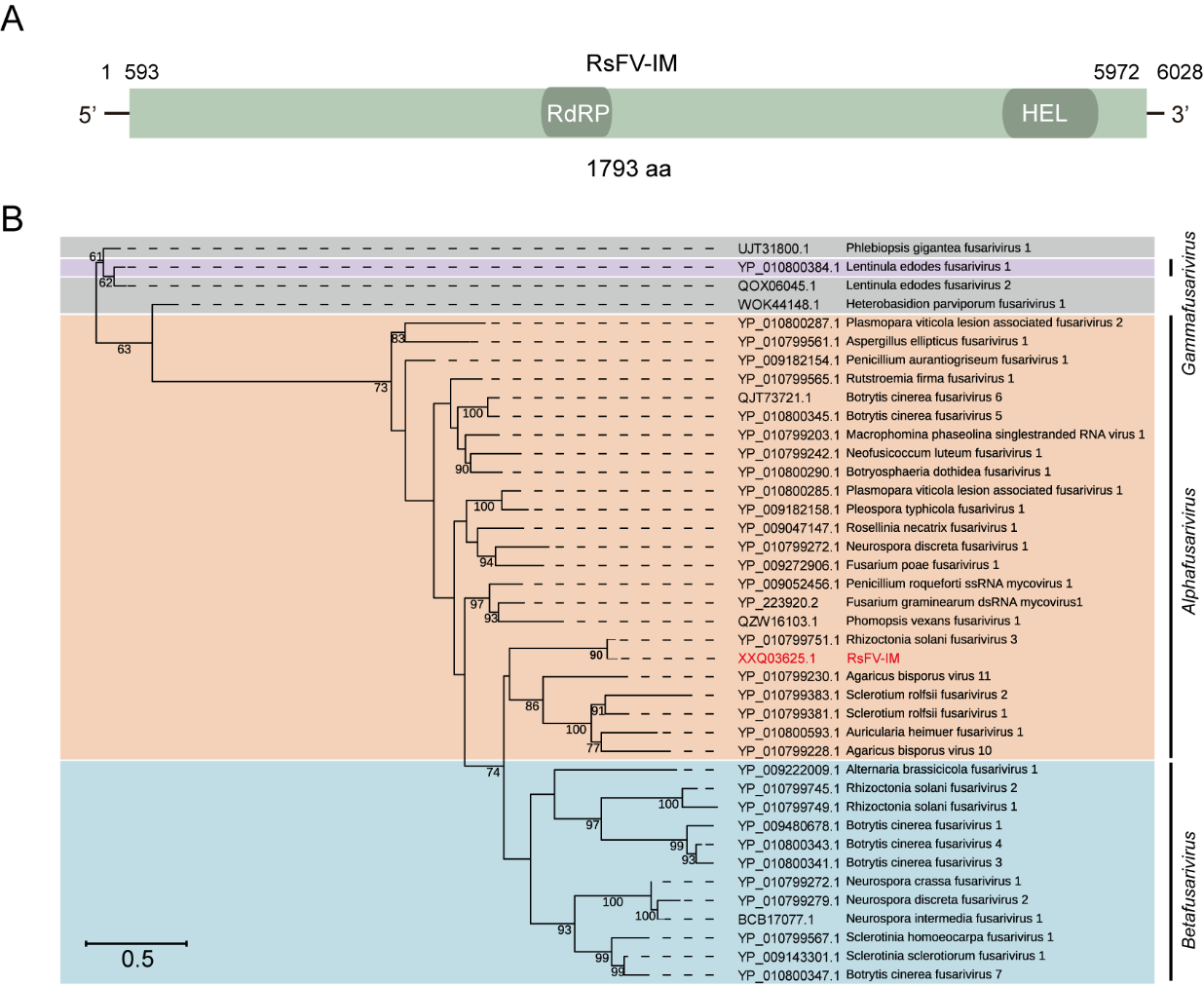


**Figure S7**. Molecular characteristics and phylogeny of RsFV-IM. (**A)** Schematic representation (not to scale) of the RsFV-IM genome structure. Colored boxes represent open reading frames (ORFs), and black lines indicate 5′- and 3′-untranslated regions (UTRs). Nucleotide positions of ORFs and UTRs are labeled. Conserved domains (RdRp and helicase [Hel]) are shown as colored capsule-shaped forms within ORFs. (**B**) Phylogenetic relationships of RsFV-IM to other fusariviruses. The tree was constructed using maximum-likelihood (JTT matrix-based model) from an RdRp domain alignment. Branch numbers indicate bootstrap support (%), and branch lengths reflect substitutions per site. Virus names include accession numbers.


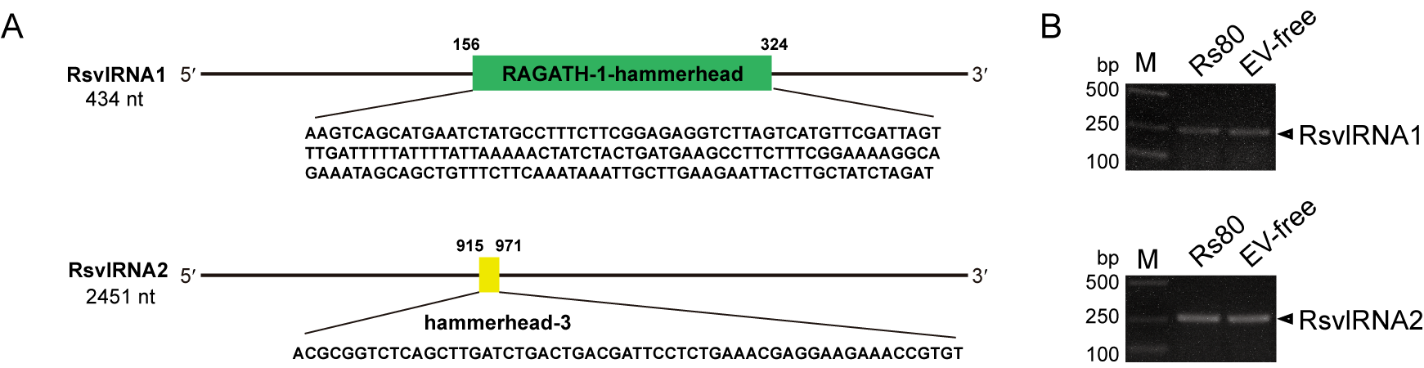


**Figure S8.** Viroid-like RNAs in *R. solani* Rs80 strain identified using VdSearch pipeline. (**A**) Predicted ribozyme sequences in viroid-like RNAs. (**B**) RT-PCR detection of viroid-like RNAs in Rs80 and EV-free strains.


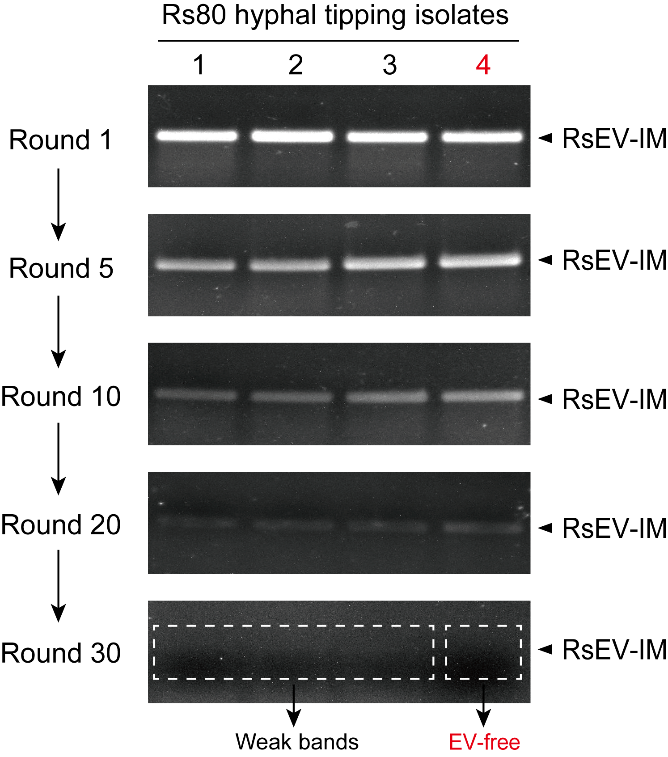


**Figure S9**. RT-PCR detection of RsEV-IM in fungal isolates derived from successive rounds of hyphal tipping.


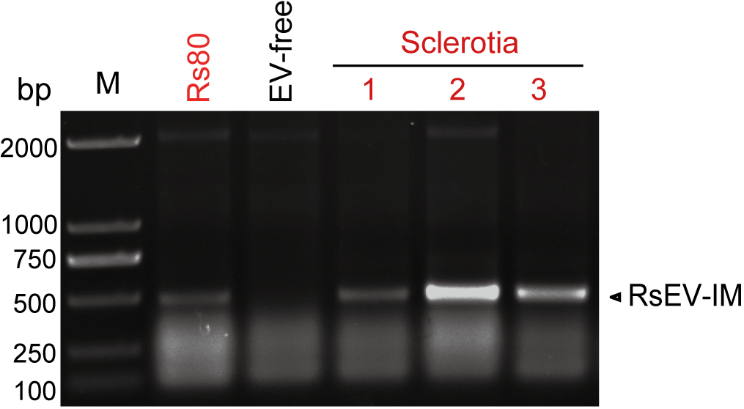


**Figure S10**. RT-PCR detection of RsEV-IM in fungal isolates regenerated from sclerotia.


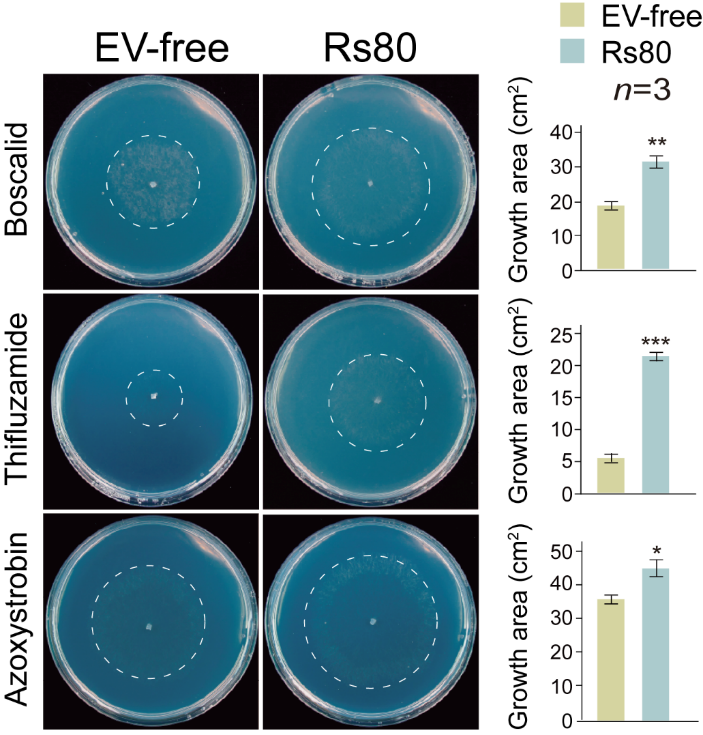


**Figure S11**. Phenotypic growth of Rs80 and EV-free strains on PDA medium (90 mm plates; imaged at 3 days) supplemented with fungicides. Colony areas (mean ± SD, *n*=3) are shown. Asterisks indicate significance (**P* < 0.05, ***P* < 0.01, ****P* < 0.001; Student’s *t*-test).


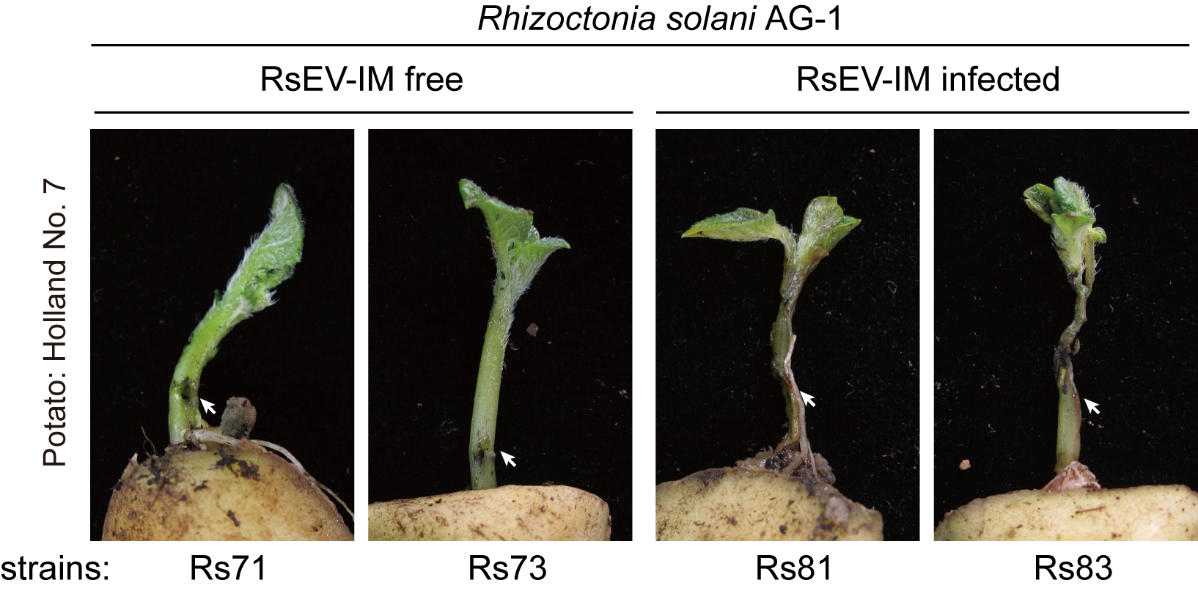


**Figure S12**. Development of stem rot in lower stems of potato shoots inoculated with RsEV-IM-infected and -free *R. solani* AG-1 strains. Plants were photographed at 7 days post-inoculation (dpi). The presented image is representative of three inoculated plants.


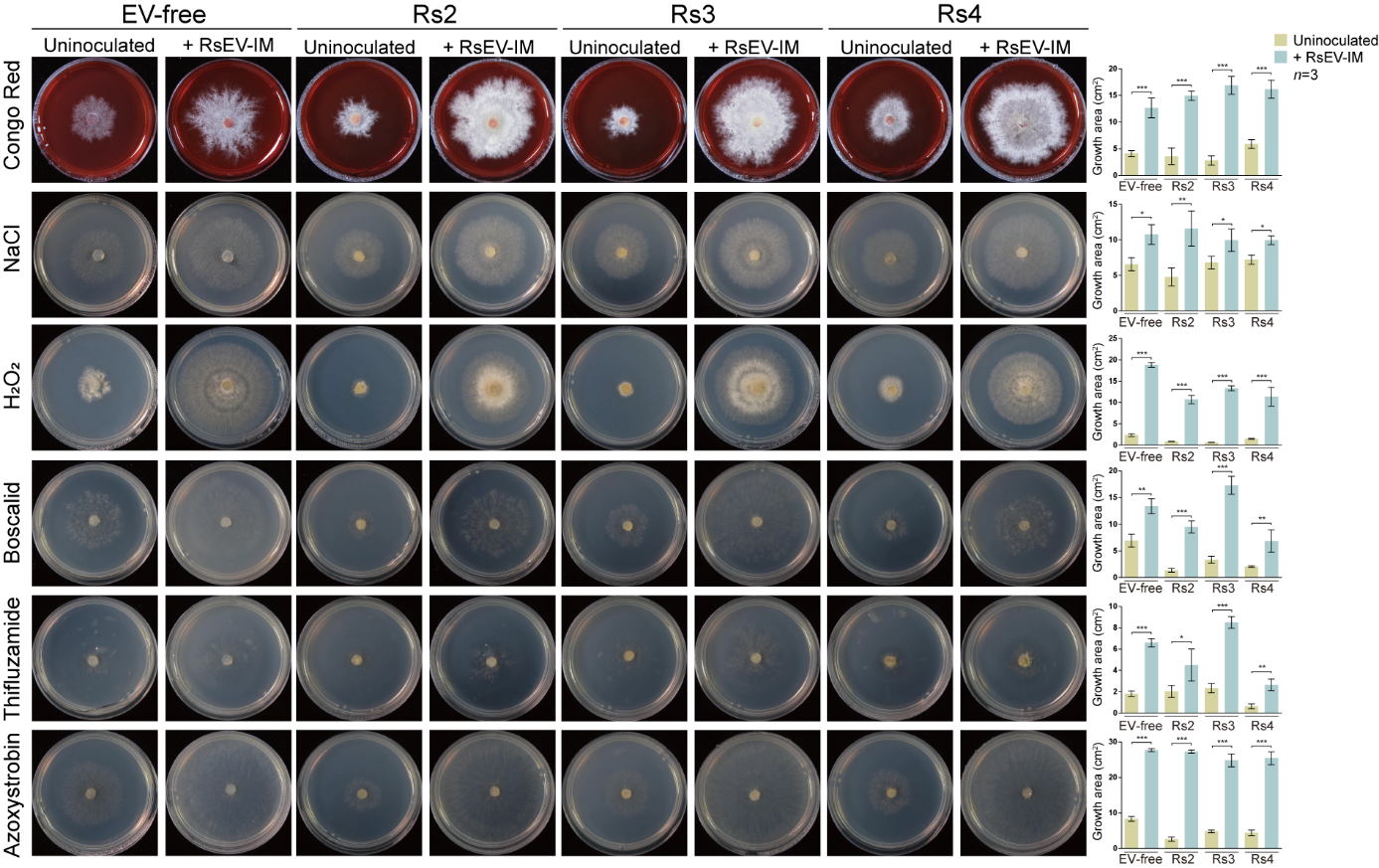


**Figure S13**. Phenotypic growth of fungal strains that had been inoculated with RsEV-IM on PDA medium (60 mm plates; imaged at 3-4 days) supplemented with stress-inducing agents (congo red; cell wall stress, NaCl; osmotic stress, H_2_O_2_; oxidative stress) and fungicides (boscalid, thifluzamide, azoxystrobin). Colony areas (mean ± SD, *n*=3) are shown. Asterisks indicate significance (**P* < 0.05, ***P* < 0.01, ****P* < 0.001; Student’s *t*-test).


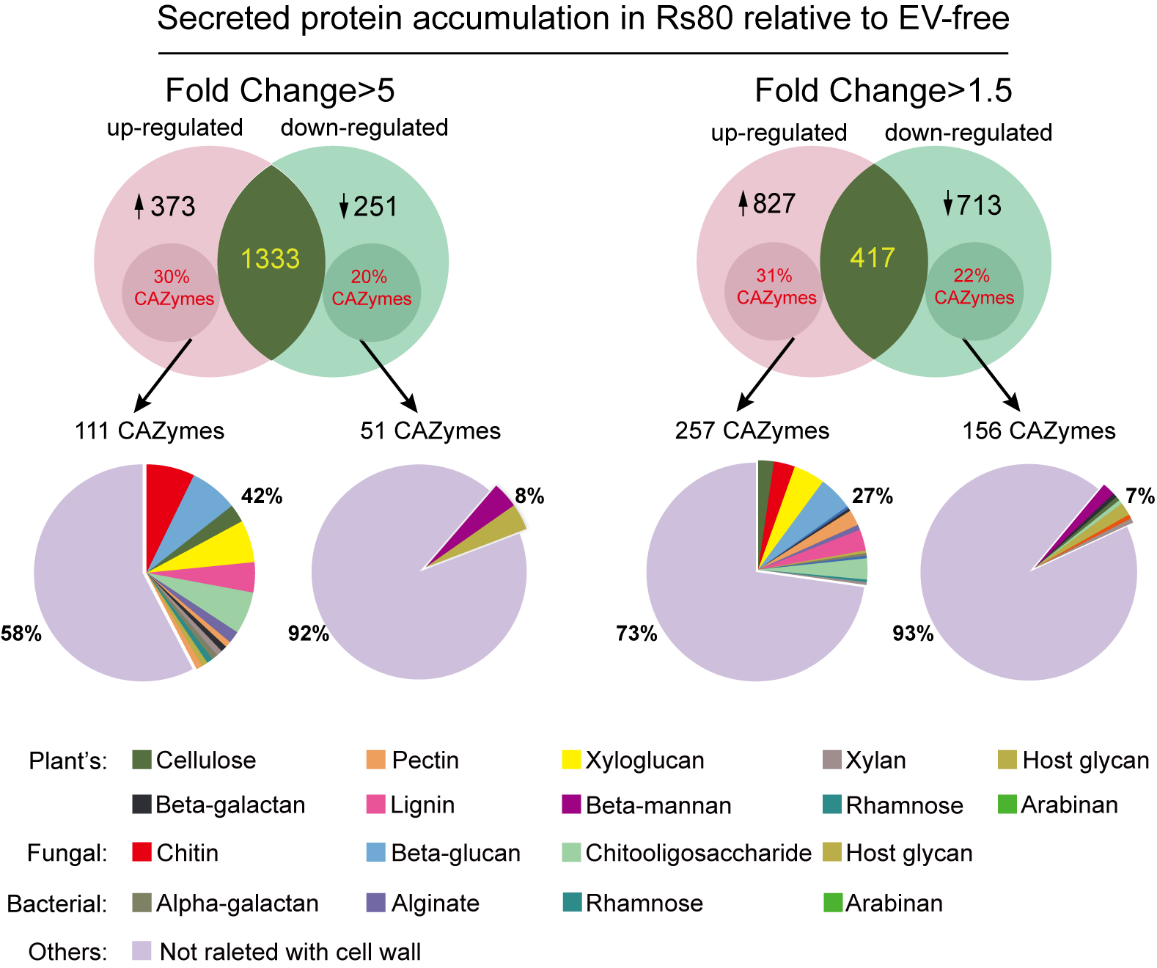


**Figure S14**. LC-MS/MS analysis of secreted protein fractions from Rs80 and EV-free strains. Proportions of differentially abundant cell wall-digesting enzymes (grouped by substrate specificity) are shown.


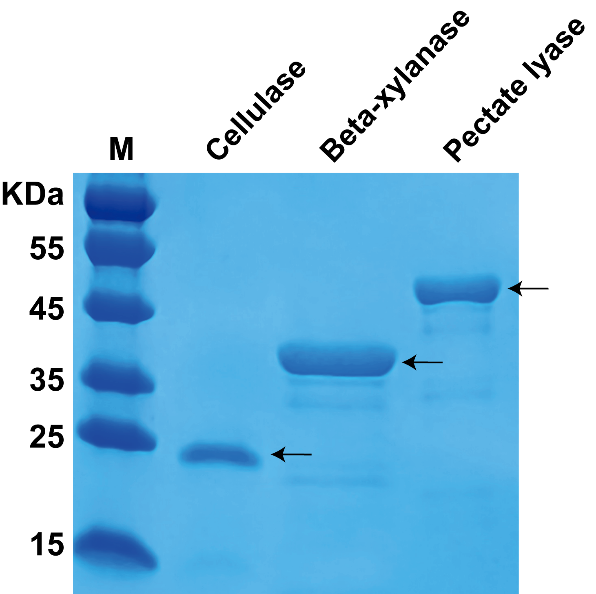


**Figure S15.** SDS-PAGE analysis of *R. solani* genes encoding cellulase, beta-xylanase, and pectate lyase expressed using yeast expression system. Arrows mark the protein bands.


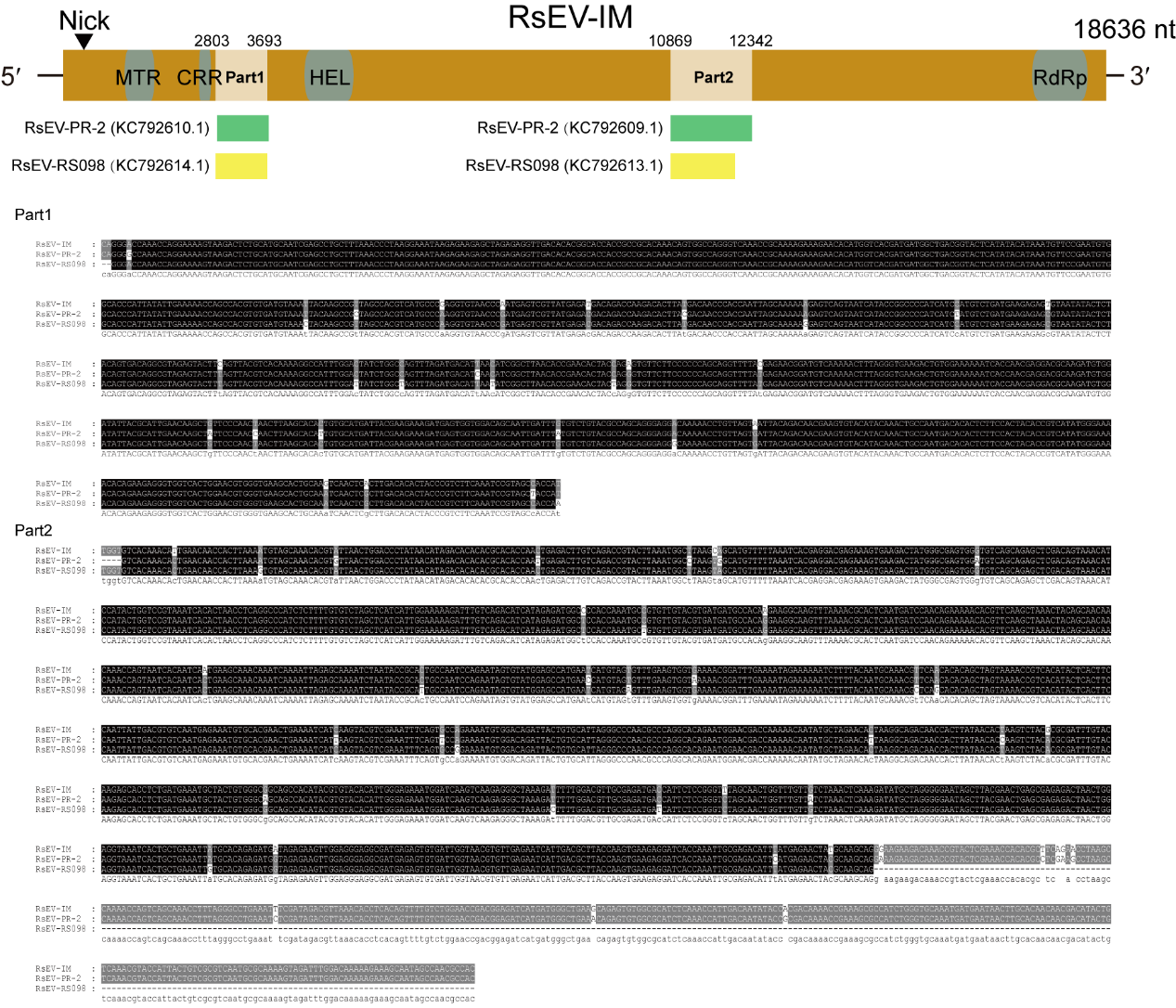


**Figure S16**. Mapping of partial sequences from dsRNA extracted from *R. solani* AG-3 strains isolated from potato plants in New Zealand to RsEV-IM genome.

**Table S1.** Severe stem rot induced by *Rhizoctonia solani* strains on various plants species.

| *R. solani* strain | Virus | | | | Source location of fungal strain | Plant species | | | | | | | |
| --- | --- | --- | --- | --- | --- | --- | --- | --- | --- | --- | --- | --- | --- |
|  | RsEV-IM | RsFV-IM | RsvlRNA1 | RsvlRNA2 |  | Potato | Potato | Tomato | *N. bentha-miana* | Pepper | Cucum-ber | Water- melon | Radish |
|  |  |  |  |  |  | (Long 3) | (Holland 7) | (Hezuo 903) |  | (Chiyan 6) | (Jin 7) | (Lanhan) | (Jiujinwang) |
| Rs1 |  | √ | √ | √ | Sizhiwangqi Banner | **-** | **-** | **-** | **-** | **-** | **-** | **-** | **-** |
| Rs2 |  |  | √ |  | Wuchuan County | **+** | **+** | **+** | **+** | **+** | **+** | **+** | **+** |
| Rs3 |  | √ | √ | √ | Wuchuan County | **-** | **-** | **-** | **-** | **-** | **-** | **-** | **-** |
| Rs4 |  | √ | √ | √ | Wuchuan County | **-** | **-** | **-** | **-** | **-** | **-** | **-** | **-** |
| Rs5 |  | √ |  | √ | Wuchuan County | **-** | **-** | **-** | **-** | **-** | **-** | **-** | **-** |
| Rs6 |  | √ |  | √ | Wuchuan County | **-** | **-** | **-** | **-** | **-** | **-** | **-** | **-** |
| Rs7 | √ | √ | √ | √ | Sizhiwangqi Banner | **+** | **-** | **+** | **-** | **+** | **-** | **-** | **-** |
| Rs8 | √ | √ |  | √ | Wuchuan County | **+** | **+** | **+** | **+** | **+** | **-** | **-** | **+** |
| Rs9 | √ |  |  | √ | Wuchuan County | **+** | **+** | **+** | **+** | **+** | **+** | **+** | **+** |
| Rs10 | √ | √ | √ |  | Wuchuan County | **+** | **+** | **+** | **+** | **+** | **+** | **+** | **+** |
| Rs11 | √ |  | √ | √ | Darhan Muminggan Joint Banner | **+** | **+** | **+** | **+** | **+** | **+** | **+** | **+** |
| Rs12 | √ | √ | √ | √ | Sizhiwangqi Banner | **+** | **+** | **+** | **+** | **+** | **+** | **+** | **+** |
| Rs13 | √ | √ | √ |  | Darhan Muminggan Joint Banner | **+** | **+** | **+** | **+** | **+** | **+** | **+** | **+** |
| Rs14 |  |  | √ | √ | Sizhiwangqi Banner | **-** | **-** | **-** | **-** | **-** | **-** | **-** | **-** |
| Rs15 |  |  | √ | √ | Sizhiwangqi Banner | **-** | **-** | **-** | **-** | **-** | **-** | **-** | **-** |
| Rs16 |  |  | √ | √ | Sizhiwangqi Banner | **-** | **-** | **-** | **-** | **-** | **-** | **-** | **-** |
| Rs17 |  | √ |  |  | Wuchuan County | **-** | **-** | **-** | **-** | **-** | **-** | **-** | **-** |
| Rs18 | √ |  |  | √ | Sizhiwangqi Banner | **+** | **+** | **+** | **+** | **+** | **+** | **+** | **+** |
| Rs19 |  | √ | √ |  | Wuchuan County | **-** | **-** | **-** | **-** | **-** | **-** | **-** | **+** |
| Rs20 |  | √ |  | √ | Sizhiwangqi Banner | **-** | **-** | **-** | **-** | **-** | **-** | **-** | **-** |
| Rs21 | √ | √ | √ |  | Wuchuan County | **+** | **+** | **+** | **+** | **+** | **+** | **+** | **-** |
| Rs22 |  |  |  | √ | Wuchuan County | **-** | **-** | **-** | **-** | **-** | **-** | **-** | **-** |
| Rs23 | √ |  |  |  | Wuchuan County | **+** | **+** | **+** | **+** | **+** | **+** | **+** | **+** |
| Rs24 |  | √ | √ |  | Wuchuan County | **-** | **-** | **-** | **-** | **-** | **-** | **-** | **-** |
| Rs25 | √ |  | √ |  | Wuchuan County | **+** | **+** | **+** | **+** | **+** | **+** | **+** | **+** |
| Rs26 |  | √ |  | √ | Sizhiwangqi Banner | **-** | **-** | **-** | **-** | **-** | **-** | **-** | **-** |
| Rs27 |  |  | √ |  | Sizhiwangqi Banner | **-** | **-** | **-** | **-** | **-** | **-** | **-** | **-** |
| Rs28 |  |  | √ | √ | Darhan Muminggan Joint Banner | **-** | **-** | **-** | **-** | **-** | **-** | **-** | **-** |
| Rs29 | √ |  | √ | √ | Wuchuan County | **-** | **-** | **-** | **-** | **-** | **-** | **-** | **-** |
| Rs30 | √ |  | √ | √ | Darhan Muminggan Joint Banner | **+** | **+** | **+** | **+** | **+** | **+** | **+** | **+** |
| Rs31 | √ | √ |  | √ | Sizhiwangqi Banner | **+** | **+** | **+** | **+** | **+** | **+** | **+** | **+** |
| Rs32 | √ | √ |  |  | Wuchuan County | **+** | **+** | **+** | **+** | **+** | **+** | **+** | **+** |
| Rs33 | √ | √ | √ | √ | Wuchuan County | **+** | **+** | **+** | **+** | **+** | **+** | **+** | **+** |
| Rs34 |  |  |  | √ | Wuchuan County | **-** | **-** | **-** | **-** | **-** | **-** | **-** | **-** |
| Rs35 |  |  |  |  | Sizhiwangqi Banner | **-** | **-** | **-** | **-** | **-** | **-** | **-** | **-** |
| Rs36 |  |  | √ |  | Darhan Muminggan Joint Banner | **-** | **-** | **-** | **-** | **-** | **-** | **-** | **-** |
| Rs37 | √ |  | √ |  | Sizhiwangqi Banner | **+** | **+** | **+** | **+** | **+** | **+** | **+** | **+** |
| Rs38 | √ |  |  | √ | Jungar Banner | **+** | **+** | **+** | **+** | **+** | **+** | **+** | **+** |
| Rs39 | √ |  | √ | √ | Sizhiwangqi Banner | **+** | **+** | **+** | **+** | **+** | **+** | **+** | **+** |
| Rs40 | √ |  |  | √ | Wuchuan County | **+** | **+** | **+** | **+** | **+** | **+** | **+** | **+** |

“√”: virus infection, “+”: plant death, “-”:small fungal lesions at the inoculation sites.

**Table S2.** A list of primers and siRNA sequences used in this study.

| Applications | Primer Name | Oligonucleotide sequence (5’- 3’) |
| --- | --- | --- |
| Primers used for PCR | | |
|  | ITS1 | TCCGTAGGTGAACCTGCGG |
|  | ITS4 | TCCTCCGCTTATTGATATGC |
| Primers used for RT-qPCR |  |  |
|  | RsEV-IM-18284F | AACGGTGTGTCAGAAGGGAG |
|  | RsEV-IM-18606R | GAACCCTCCCTCGTCCTTT |
|  | *R.solani*-β-tubulin-F | CACTCACTCTCTTGGTGGTGG |
|  | *R.solani*-β-tubulin-R | CGTTGTCGATGCAGAATGTCTC |
| Primers used for RT-PCR/RACE | | |
|  | RsEV-IM-5UTR-RACE-R | GGCAAGTATGATTTTCCTCTCTG |
|  | RsEV-IM-3UTR-RACE-F | CTAGATCGATGAGTTATGCCATG |
|  | RsEV-IM-23F | GCAAACACTCACCACATCCCT |
|  | RsEV-IM-460R | TTCTGGCTCCGCTATTTCTGT |
|  | RsEV-IM-9041F | GCTTATGCGAGTTGGTTGTC |
|  | RsEV-IM-9676R | CATTCTCACCGTCTTCCGATC |
|  | RsEV-IM-5112F | ATGGTTGGCAGTCAACACTTGTGTG |
|  | RsEV-IM-5626R | TCCGCTCATTTGTGAGACCT |
|  | RsFV-IM-F | TCGCACCTGAATGGCTGTC |
|  | RsFV-IM-R | AAAGTCGTGAATGAGGGAGTC |
|  | RsvlRNA1-F | CTGCAGTCCAAGTCCATGTT |
|  | RsvlRNA1-R | TCTGCCTTTTCCGAAAGAAGG |
|  | RsvlRNA2-F | ATAAGTCGATAGGGCTTGGAG |
|  | RsvlRNA2-R | GCTGGTCCAGCCTTAATATTC |
| Primers used for plasmid construction |  |  |
|  | pYES2-RSOL_306800-F | CTATAGGGAATATTAAGCTTATGAAATTCGCTATTTCTCT |
|  | pYES2-RSOL_306800-R | ACTGGCGGCCGTTACTAGTGGATCCAGCGTAATCTGGAACAT  CGTAGGGTACTTTGCGAGAGCACCCAGTGAT |
|  | pYES2-RSOL_512310-F | CTATAGGGAATATTAAGCTTATGGTCACTTCTCCCCGCC |
|  | pYES2-RSOL_512310-R | ACTGGCGGCCGTTACTAGTGGATCCAGCGTAATCTGGAACATCGTAGGGTACGGCAAGAGCCTGAGCAACGG |
|  | pYES2-RSOL_396370-F | CTATAGGGAATATTAAGCTTATGGTTGGATTCAAGTCGAC |
|  | pYES2-RSOL_396370-R | ACTGGCGGCCGTTACTAGTGGATCCAGCGTAATCTGGAACATCGTAGGGTACAAGTTTCCCAGCACCCGCGTT |
| Primers used for Northern blot | | |
|  | RsEV-IM-Nblot-RdRp-F | ACAACAAGGAGTGGAAGTAAACC |
|  | RsEV-IM-Nblot-RdRp-R | CGCATAGCGTCACCGTATC |
| siRNA sequences | | |
|  | siRNA-ns | UUCUCCGAACGUGUCACGU(dT)(dT) |
|  | siRNA-RsEV-IM-HEL | UCUAUCAUGUUAACGUCUA(dT)(dT) |
|  | siRNA-RsEV-IM-MTR | UUGAACACUUUGUACACAC(dT)(dT) |
|  | siRNA-RsEV-IM-RdRp | ACAUGAACAUGUUGUCAUC(dT)(dT) |
